# Unusual complication and successful high-dose chemotherapy treatment of advanced Burkitt’s lymphoma in an adult HIV-positive patient

**DOI:** 10.4102/sajr.v21i2.1230

**Published:** 2017-11-14

**Authors:** Henrietta W.H. McGrath, Alexander Fitzhugh, Maria Javed, Neesha Rockwood, Farhat Kazmi

**Affiliations:** 1 Department of Radiology, Chelsea and Westminster Hospital, United Kingdom; 2 Department of Infectious Diseases and HIV, Chelsea and Westminster Hospital, United Kingdom

## Abstract

Adult Burkitt’s lymphoma emerged as an AIDS-defining condition in the 1980s. We describe a case of HIV-associated adult Burkitt’s lymphoma diagnosed and treated with high-dose chemotherapy in our institution, complicated by unusual bilateral renal vein tumour thrombi and tumour lysis syndrome. We believe this unique case highlights the need for early recognition of current and potential complications on staging computed tomography imaging, as well as successful use of a high-dose chemotherapy regimen.

## Introduction

Burkitt’s lymphoma is a highly proliferative B-cell neoplasia first described as a childhood illness endemic to malaria areas of Africa. Following the emergence of HIV in the 1980s, its significance as an AIDS-defining condition was soon recognised with a far more aggressive natural history, and its significance as an AIDS-defining condition was demonstrated.

HIV-associated lymphomas remain a significant cause of morbidity and mortality, especially within sub-Saharan Africa, and familiarity with imaging findings is essential in suggesting the initial diagnosis, identifying complications and assessing response to treatment. We present a case of adult Burkitt’s lymphoma with advanced disease.

## Case report

A 49-year-old man presented near cardiac-arrest with a 4-week history of weight loss, night sweats, back pain and difficulty walking.

**FIGURE 1:**
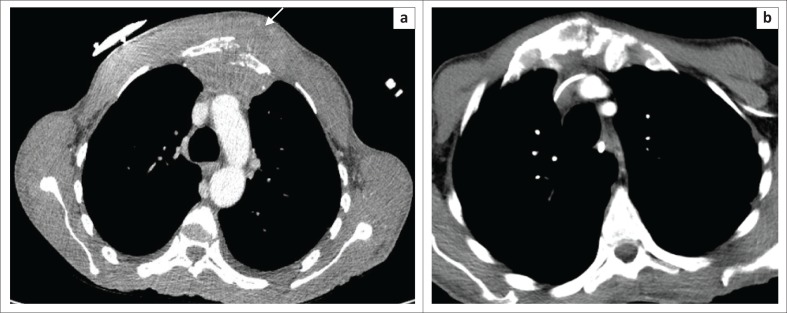
(a) Axial post-contrast CT imaging of the thorax on soft tissue windows demonstrates destruction of the sternum with surrounding soft tissue mass lesion (arrow); (b) Post-contrast imaging 1 month later demonstrates a good response to treatment, with marked reduction in soft tissue volume and bony infiltration.

He was cachectic (42 kg), with massive hepatomegaly and a large sternal soft tissue mass (Figure 1). Blood tests demonstrated acute renal failure (creatinine 168 µmol/L, lactate 22 mmol/L, calcium 2.84 and phosphate 3.1 mmol/L).

A new HIV-1 diagnosis was made (CD4 lymphocyte count 3 cells/µL, viral load 912 376 copies/mL). Epstein-Barr viral titres were 21 620 500 copies/mL.

**FIGURE 2:**
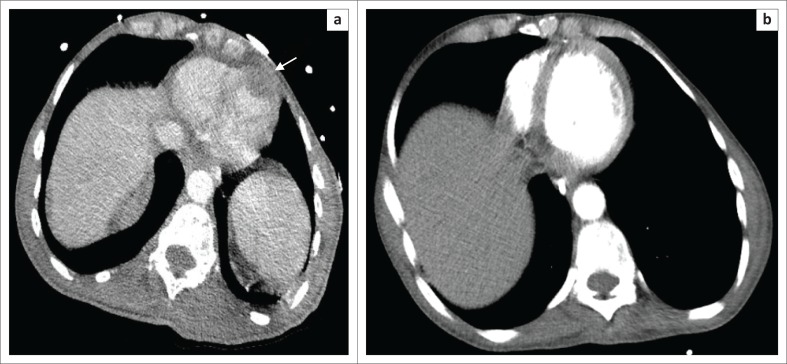
(a) Axial post-contrast thoracic CT imaging demonstrates pericardial tumour infiltration (arrow) with full resolution on (b) post-contrast imaging obtained 1 month later.

Computed tomography (CT) suggested Burkitt’s lymphoma with disease affecting the lungs, heart (Figure 2), sternum, liver, vertebrae and kidneys with bilateral renal vein tumour thrombi. Biopsy of the sternal mass demonstrated diffuse lymphoid infiltrates of skeletal muscle in a starry sky pattern, in keeping with Burkitt’s lymphoma.

The patient was commenced on antiretroviral therapy including Descovy and Dolutegravir, with a Rituximab-CODOX-M/IVAC chemotherapy regimen.^1^

His initial management was complicated by tumour lysis syndrome,^2^ pseudomonas bacteraemia and cytomegalovirus infection of the gastrointestinal tract, all successfully treated.

Subsequent CT imaging revealed an excellent treatment response, regression of extensive hepatic and renal infiltration and resolution of bilateral renal vein tumour thrombi, allowing anticoagulation to be discontinued.

## Discussion

First reported in 1958, Burkitt’s lymphoma can be usefully subdivided into endemic, sporadic (without geographical limitation) and immunodeficiency-associated. Predominantly a disease of childhood (representing 40% of paediatric non-Hodgkin’s lymphomas^3^), Burkitt’s lymphoma represents fewer than 5% of lymphoma affecting adults and carries a worse prognosis with advancing age (5-year survival between 50% and 65% without central nervous system [CNS] involvement at presentation^3^). Our patient presented at the age of 49 with CT-stage IV HIV-associated immunodeficient Burkitt’s lymphoma, with a high lactate on admission, an additional poor prognostic marker (2-year survival of 63% vs. 86% of patients with a normal serum lactate level).^3^

**FIGURE 3:**
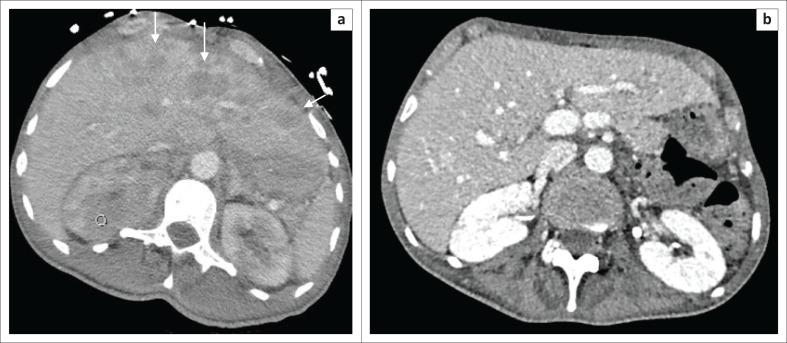
(a) Axial post-contrast abdominal CT demonstrates extensive liver tumour infiltration (arrows) with compression of the left portal vein. Right renal tumour infiltration is illustrated (circle). (b) Post-contrast imaging obtained after 1 month demonstrates marked improvement, with resolution of the left portal vein compression.

While tissue diagnosis remains essential, CT staging (using both St Jude/Murphy’s and Ann Arbor scoring systems^4^) guides treatment. Early recognition of extensive intra-abdominal involvement [renal and hepatic (Figure 3)] helped the clinicians anticipate and promptly treat tumour lysis syndrome. Most centres commence treatment within 24 h – 48 h, and CT should not delay pursuit of a tissue diagnosis or treatment.

**FIGURE 4:**
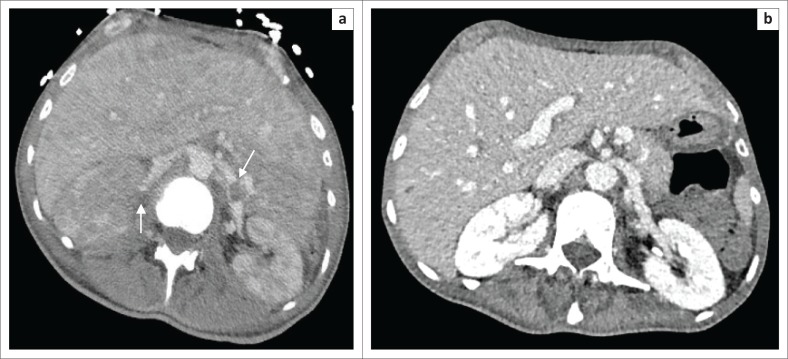
(a) Axial post-contrast abdominal CT demonstrates bilateral renal vein tumour thrombi (arrows) with extensive renal tumour infiltration, most marked on the right. The tumour thrombi have resolved on (b) post-contrast imaging obtained 1 month later, and there has been a marked reduction in renal tumour volume.

Bilateral renal vein tumour thrombosis is an uncommon complication, not typically seen in lymphoma, necessitating anticoagulation (Figure 4).^5,6^ CT confirmed resolution, allowing a return to thromboprophylaxis alone.

Disease confined to the abdomen is described in 50%,^7^ usually hepatosplenomegaly which may be observed on ultrasound. Abdominal and pelvic CT is beneficial for further characterisation and better demonstrates potential involvement of the distal small bowel, caecum and appendix.^8^

Involvement of the central nervous system heralds a poor prognosis (5-year survival drops to < 30%); our patient underwent CT and magnetic resonance imaging (MRI) brain which did not demonstrate CNS disease. Bilateral cavernous sinus involvement has been described in an immunocompetent individual with confirmed Burkett’s lymphoma,^9^ diagnosed using CT and multi-parametric MRI; although the patient responded to first line cyclophosphamide-vincristine chemotherapy, CNS disease recurred and the patient died after 11 months.

Involvement of the distal urinary tract, testicular involvement, pancreatic disease and jaw involvement has been described in adults. Positron emission tomography–computed tomography (PET-CT) has been utilised to identify additional sites of disease, with a low false negative rate. However, a high false positive rate has been noticed within the immunosuppressed population attributed to concurrent opportunistic infections.

## Conclusion

We have described the imaging findings of Burkitt’s lymphoma affecting an immunodeficient adult, emphasising the need for prompt diagnosis. CT delineated our patient’s disease, subsequently confirming an excellent response to high-dose chemotherapy, and identified current (renal vein tumour thrombus) and potential complications (tumour lysis syndrome). Radiologists should employ alternative modalities (ultrasound and MRI) to investigate extra-abdominal disease foci where clinically appropriate.
